# First Human Results With the 256 Channel Intelligent Micro Implant Eye (IMIE 256)

**DOI:** 10.1167/tvst.10.10.14

**Published:** 2021-10-27

**Authors:** Huizhuo Xu, Xingwu Zhong, Changlin Pang, Jing Zou, Wangling Chen, Xianggui Wang, Shanxiang Li, Yuntao Hu, Didier S. Sagan, Philip T. Weiss, Yangyi Yao, Jiayi Xiang, Margot S. Dayan, Mark S. Humayun, Yu-Chong Tai

**Affiliations:** 1 Department of Ophthalmology, Xiangya Hospital, Central South University, Changsha, Hunan Province, China; 2 Hainan Eye Hospital and Key Laboratory of Ophthalmology, Zhongshan Ophthalmic Center, Sun Yat-Sen University, Haikou, Hainan Province, China; 3 USC Ginsburg Institute for Biomedical Therapeutics, USC Roski Eye Institute, Department of Ophthalmology and Biomedical Engineering, University of Southern California, Los Angeles, CA, USA; 4 Departments of Electrical Engineering and Bioengineering, California Institute of Technology, Pasadena, CA, USA; 5 Golden Eye Bionics, LLC, Pasadena, CA, USA; 6 IntelliMicro Medical Co., Ltd., Changsha, Hunan Province, China; 7 Department of ophthalmology, Beijing Tsinghua Changgung Hospital, Tsinghua University, Beijing, China; 8 North Hollywood Senior High School, North Hollywood, CA, USA

**Keywords:** retinitis pigmentosa, retinal degeneration, retinal prosthesis, visual rehabilitation, blindness

## Abstract

**Purpose:**

To report on the safety and efficacy of the 256-channel Intelligent Micro Implant Eye epiretinal prosthesis system (IMIE 256).

**Methods:**

The IMIE 256 implants were implanted in the right eyes of five subjects with end-stage retinitis pigmentosa. Following implantation, the subjects underwent visual rehabilitation training for 90 days, and their visual performance was evaluated using the grating visual acuity test, Tumbling E visual acuity test, direction of motion, square localization, and orientation and mobility test. To evaluate the safety of the IMIE 256, all adverse events were recorded.

**Results:**

Subjects performed significantly better on all evaluations with the IMIE 256 system on as compared with the performance at baseline or with the system off. There was a steady improvement in performance at each observation interval, indicating that the training and/or practice helped the subjects use the IMIE 256. There were two serious adverse events—electrode array movement and low intraocular pressure in one subject, which resolved with surgery. There were no other adverse events observed except those expected in the course of postoperative healing.

**Conclusions:**

These results show an improved safety and efficacy profile compared with that of the Argus II implant. Further clinical trials are needed to confirm these results in a larger number of subjects and over longer durations.

**Translational Relevance:**

To our knowledge, this study reports the first in-human data from a high-density (256 electrodes) epiretinal implant to restore sight to a subset of blind patients.

## Introduction

By bypassing damaged and degenerated photoreceptors and stimulating remaining retinal neurons, retinal prosthetics such as the Argus II (Second Sight Medical Products, Sylmar, CA) have successfully restored some degree of sight to those blinded by retinitis pigmentosa.^1^^–^^6^ However, the visual acuity of current devices is limited, and some of the contributing factors are high stimulation threshold and low electrode density.^7^ The 256-channel Intelligent Micro Implant Eye (IMIE 256) is an epiretinal prosthesis system also referred to as the Theia α Implantable Retinal Stimulator. It was co-developed by Golden Eye Bionic, LLC (Pasadena CA) and IntelliMicro Medical Co., Ltd. (Changsha, Hunan Province, China) and is manufactured by IntelliMicro. The IMIE 256 seeks to address some of the challenges current retinal prosthetics are facing. This study presents the physiological and functional results of visual tests conducted for 90 days following implantation of the IMIE 256 in five subjects and compares them to the published results of the Argus II retinal prosthesis system.

## Methods

### Statement of Compliance

This clinical study was conducted per Measures for the Management of Clinical Research Projects Carried out by Medical and Health Institutions (2014, No. 80), announcement by the National Health Commission of the People's Republic of China, China Food and Drug Administration, and the National Administration of Traditional Chinese Medicine of the People's Republic of China. The study was approved by the Medical Ethics Committee of Xiangya Hospital Central South University; the Medical Ethics Committee of Hainan Eye Hospital and Key Laboratory of Ophthalmology, Zhongshan Ophthalmic Center, Sun Yat-Sen University; the Health Commission of Hunan Province; and the Health Commission of Hainan Province, People's Republic of China. All subjects signed an informed consent prior to participating in this study after the nature and risks of this study were explained.

### Device Description

**Figure 1.**
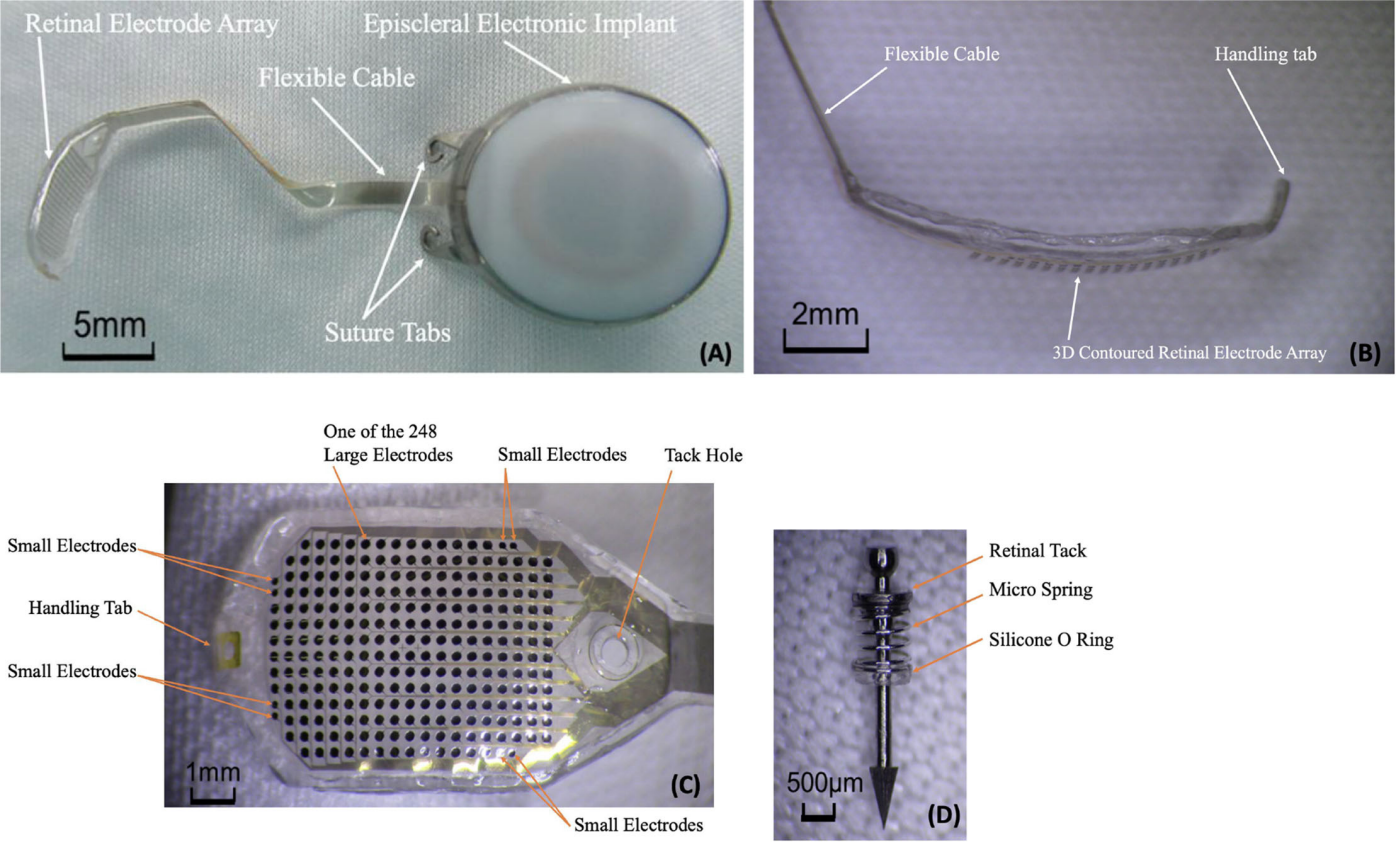
IMIE 256 implantable device. (A) Implant (*upper left*), (B) side view of the retinal electrode array (*upper right*), (C) top view of the retinal electrode array (*lower left*), and (D) retinal tack (*lower right*).

**Figure 2.**
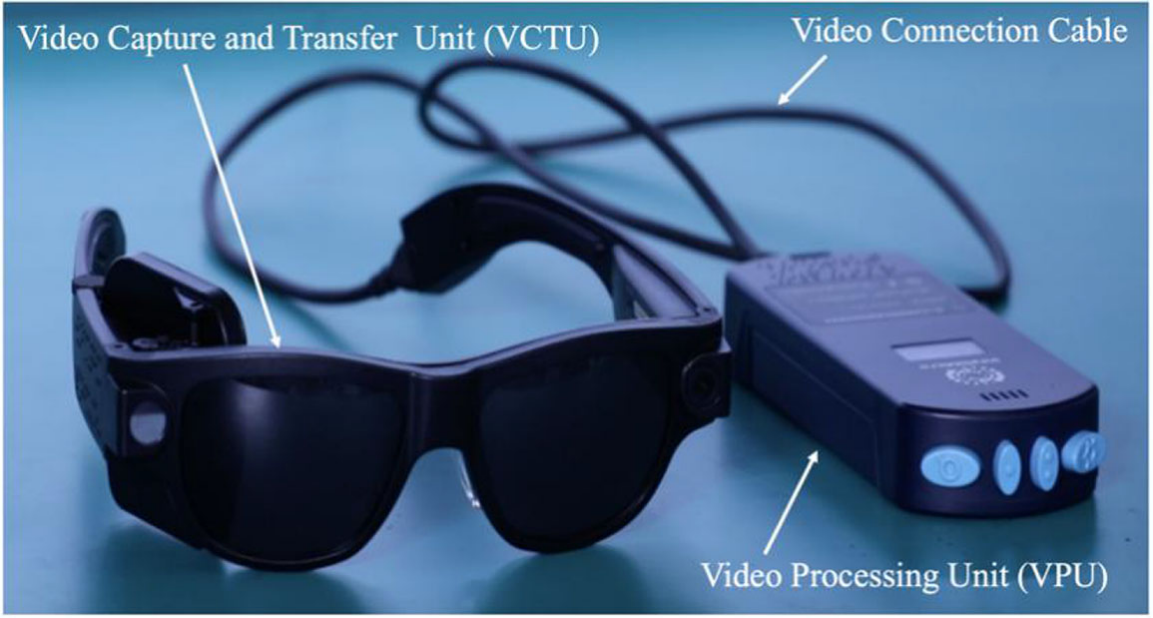
IMIE 256 VCTU and VPU.

**Figure 3.**
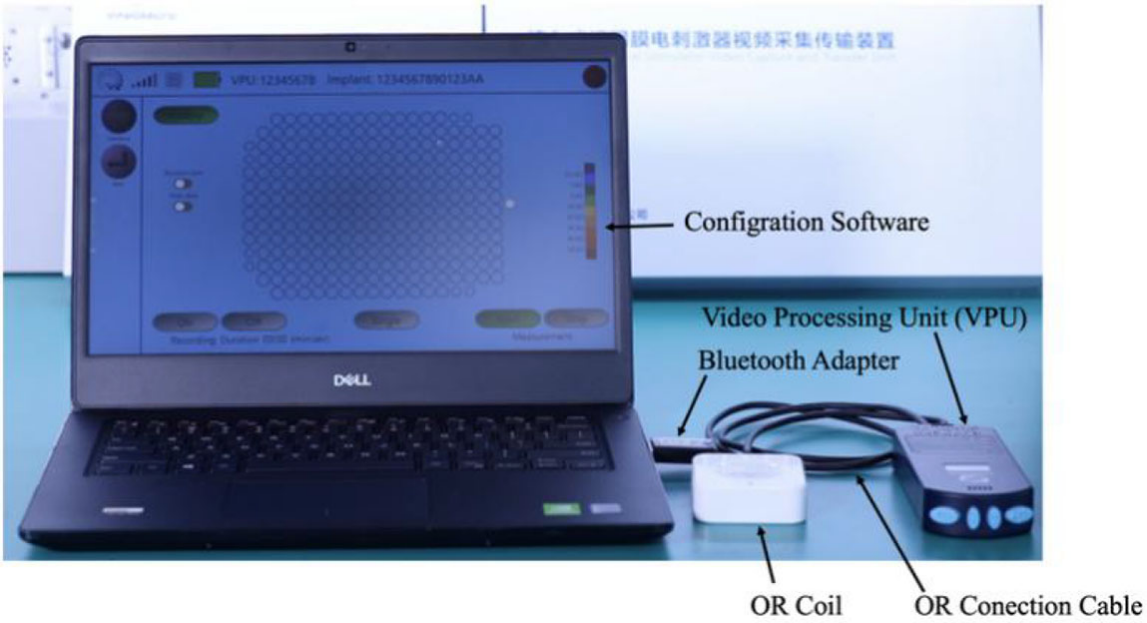
IMIE 256 clinical fitting/configuration system. The screen shows individual electrodes; the system allows current settings for each electrode for each subject.

The IMIE 256 consists of four subsystems, including an implantable device (Fig. 1); a video capture and transfer unit (VCTU) and a video processing unit (VPU) (Fig. 2); and a clinical fitting/configuration system (Fig. 3). The implant (Fig. 1A) includes an episcleral electronic implant, a trans-scleral microfabricated flexible cable, a custom contoured retinal electrode array (Fig. 1B) that consists of a total of 256 electrodes covering an area of 4.75 mm × 6.50 mm (corresponding to a visual field of 14.7° of height and 20.1° of width), with two sizes of disc-shaped electrode diameters: 248 large electrodes (210 µm in diameter) and 8 smaller electrodes (160 µm in diameter) (Fig. 1C). The center-to-center pitch is 350 µm for the large electrodes and 300 µm for the small electrodes. The purpose of the smaller electrodes is to verify the stimulation capability of such electrodes to evaluate the feasibility of a next generation epiretinal prostheses with 512 or 1024 electrodes. The electrode surface material is platinum gray, and the insulating coating material is Parylene C. The theoretical resolution limit of the electrode array area with the large electrodes is 1.08°. For both large and small electrodes, the maximum safe stimulation charge density is 0.29 mC/cm^2^ for continuous use and 1 mC/cm^2^ for short-term use when testing the subject's stimulation perception threshold and saturation current. For continuous stimulation, the stimulation pulse amplitude and the charge-per-pulse maximum were set at 200 µA and 90 nC for the large electrodes and 116 µA and 52 nC for the small electrodes, respectively. The electrode array is affixed to the retina with a custom retinal tack (Fig. 1D). See Supplementary Material S1 for the details of device description.

### Subject Selection

Five subjects who met the inclusion and exclusion criteria shown in Table 1 were implanted with the IMIE 256 implant. For each subject, a complete ophthalmology examination was performed that included determining intraocular pressure (IOP) and best-corrected visual acuity, slit-lamp examination, fundus photography, ultrasound A- and B-scans, optical coherence tomography (OCT), electroretinograms (ERGs), visual evoked potentials, flashlight test, and electrical evoked responses (EERs). An ultrasound A-scan was used to measure the axial length of the eye, and an ultrasound B-scan was used to measure the radius of curvature of the macular region. The right eye was the worse seeing eye in four subjects and equivalent in one subject; hence, it was selected as the eye to receive the implant. The axial length and the retinal radius of curvature for the five implanted eyes ranged from 22.85 to 24.66 mm and 11.46 to 12.45 mm, respectively. EER was used to evaluate the inner retinal function of each subject. A commercially available neurostimulator (Digitimer DS7A/DG2A; Digitimer Ltd., Welwyn Garden City, Hertfordshire, UK) with a sterile single-use ERG jet electrode was used for the EER test. The EER test procedure was as follows: The cornea was anesthetized with topical anesthesia (oxybuprocaine hydrochloride eye drops; Santen Pharmaceutical, Osaka, Japan), and the disposable ERG jet electrode (Fabrinal Eye Care, Neuchâtel, Switzerland) was placed on the eye with hypromellose eye drops (Goniosol; Shenyang Xingqi Pharmaceutical, Shenyang, China). An ear-clip EEG cup electrode (Technomed, Maastricht Airport, The Netherlands) was clamped on the ear lobe with electrode gel (Spectra Gel; Diagnosys LLC, Lowell, MA) and served as the ground/return electrode. The non-tested eye was patched. Testing used monophasic pulses and commenced with the stimulation amplitude set at 1 mA with a pulse width of 1 ms. Both parameters were then increased until the subject perceived an electrically elicited flash of light (phosphene). The phosphene threshold current was reached when the subject could see a phosphene at least five times out of eight stimulation pulses. The current used did not exceed 8 mA, and the pulse width did not exceed 2 ms. At no point did any of the subjects experience any discomfort other than a mild tingling sensation. Baseline data including the EER test results for the subjects are summarized in Table 2.

**Table 1.** Inclusion and Main Exclusion Criteria


Inclusion criteria	1. Adults 18 years of age and above
	2. Diagnosed with end-stage retinitis pigmentosa
	3. History of being able to read letters
	4. Either aphakic or pseudophakic or willing to have the crystalline lens removed without intraocular lens implantation
	5. Willing to accept the clinical follow-up after implantation, including vision rehabilitation
Main exclusion criteria	1. Any uncontrolled disease, which may affect the preoperative examinations, implantation operation, and postoperative follow-ups
	2. Eye diseases that may affect the implantation or functionality of the IMIE 256 implant (e.g., glaucoma, optic nerve disease, central retinal artery or vein occlusion, history of retinal detachment, trauma, severe strabismus)
	3. Eye pathology that may prevent the successful implantation of the IMIE 256 implant or postoperative healing (e.g., extremely thin conjunctiva, corneal ulcer, axial length < 20.5 mm or > 26 mm)
	4. Eye pathology that prevents full observation of the internal structure of the eyeball (such as corneal opacity, except cataract)
	5. Intolerance to general anesthesia or recommended antibiotics and steroids related to implant surgery
	6. Metal or active electronic implants in the head that may affect the normal function of the IMIE 256 implant

**Table 2.** Baseline Data for the Subjects

		Preoperative Vision						
Subject	Clinical Diagnosis	OD (Implanted Eye)	OS (Non-Implanted Eye)	EER (mA)	Age (yr)	Sex	Ethnicity	Inheritance Pattern
P01	Binocular retinitis pigmentosa	LP/10 cm (dimmer than OS)	LP/10 cm	2.5	60	Female	Chinese	Autosomal recessive
P02	Usher syndrome	LP/20 cm (dimmer than OS)	LP/20 cm	3	56	Female	Chinese	Sporadic
P03	Binocular retinitis pigmentosa	LP/3 m	LP/2 m	2	56	Female	Chinese	Sporadic
P04	Binocular retinitis pigmentosa	NLP	NLP	7–8	57	Female	Chinese	Sporadic
P05	Binocular retinitis pigmentosa	LP/5 m (dimmer than OS)	LP/5 m	4.5	54	Male	Chinese	Sporadic

EER was tested with a 2-ms pulse width. OD, right eye; OS, left eye; LP, light perception; NLP, no light perception.

### Surgical Procedure

The implantation procedure has four stages: surgical preparation, extraocular placement, intraocular placement, and closure. The electrical performance of the implant device was checked during each stage of the operation to ensure it remained functional. The explantation surgical procedure was used to remove the implant after 90 days of implantation. See Supplementary Material S2 for the details of the implantation and explantation surgical procedures and Supplementary Material S3 for preoperative, perioperative, and postoperative medications.

### Clinical Safety Evaluation

Postoperative clinical evaluations were conducted to document adverse events and serious adverse events (SAEs). SAEs were defined as causing death, being life-threatening, causing permanent damage, or necessitating surgical or medical intervention. Results of postoperative clinical evaluations were tabulated for safety analysis.

### Electrode Impedance and Threshold Current Measurement

The electrode impedance was calculated from waveform sampling measured through reverse telemetry in the operating room and at postoperative time points using the configuration system. Biphasic pulses (0.45-ms cathodic pulse followed by 0.45-ms anodic pulse with 0.05-ms interphase gap) at 1-Hz stimulation frequency were used for the threshold current measurement. The electrodes were tested individually by increasing the stimulation current until the subject reported seeing flashing phosphenes. Reliability of the subjects’ responses was verified by making sure subjects responded to three of three threshold stimulations, and when this threshold was met the current was decreased to make sure the subject could not perform at the same level at a lower current. The threshold current and the subjective characteristics of the phosphene perception were recorded and used to generate a complete threshold map for the electrode array. Fifteen current levels were used to represent level 0 to 14 grayscale brightness levels for each electrode.

### Visual Rehabilitation Training

The rehabilitation training was held at the site of IntelliMicro, an affiliated training center of Hunan Xiangya Hospital. Training started 2 weeks after implantation and lasted 90 days, with 2-hour sessions per day, 3 or 4 days per week. The training was a mix of on-screen, laboratory setting, and real world, depending on the different training purposes. Six visual rehabilitation training exercises were used.

The on-screen training included light-source positioning training, in which subjects identified the position of a light source in the field of view, and light direction of movement training, in which subjects were asked to identify the position or moving direction of a light source moving at varying speeds in the field of view on a screen with 40-cm viewing distance. Pattern/number/letter recognition training, in which subjects were asked to identify the white pattern/number/letter placed on a black background wall, and socks sorting training, where subjects were asked to classify black and white socks on a black table, were conducted in a high-contrast training room. Indoor obstacle avoidance training was conducted in a high-contrast laboratory setting with straight black lines on the floor serving as the target walking path and white foam cones as the obstacles. Subjects were asked to walk along the black lines and to avoid the foam cones on or beside the track line. Outdoor walking training was conducted in a real-world settings. With sufficient safety precautions in place to prevent falls or other accidents, subjects were asked to walk along the edge of the road and use a crosswalk.

### Clinical Efficacy Evaluation

Five tests were used to evaluate clinical efficacy: (1) grating visual acuity, in which subjects verbally described the orientation of black and white bars on a screen with the resolution of the bar set at 20/800 (0.46-cm bar width and spacing with 40-cm viewing distance); (2) Tumbling E visual acuity, which tested subjects’ ability to recognize the orientation of a Tumbling E set at 20/1200 (10.5-cm letter size with 1.2-m viewing distance); (3) direction of motion, in which subjects tracked a moving light bar (3.5 cm wide with 40-cm viewing distance) on a black screen (32 cm × 60 cm) to determine whether visual perception and direction were accurate; (4) square localization, which tested the accuracy of subjects’ ability to locate a light-emitting square (7.0 cm × 7.0 cm with 40-cm viewing distance) on a black screen (32 cm × 60 cm) by having the subjects use their finger to touch the screen to indicate the square locations (touching inside the square area was judged as correct and touching outside the square area was judged as incorrect, with a chance level of (7 × 7)/(32 × 60) = 2.6%); and (5) orientation and mobility test, which evaluated subjects’ ability to track lines (0.4 m wide and 8 m long) on the floor in the line task and their ability to locate a door (0.9 m × 2 m), which was 8 m away, in the door task. For the line task, the total line distance was 8 m; if the subjects could walk along the straight line for at least 5 m in each trial, their effort was marked correct; otherwise, incorrect. For the door task, if the subjects could walk toward the door and touch or enter the door at the end of each trial, their effort was marked as correct; otherwise, incorrect.

The grating visual acuity, Tumbling E visual acuity, and direction of motion tests were forced choice, with the number of alternatives shown in Table 3. The number of trials varied according to the subjects’ health condition and willingness to cooperate. If the subject was unable to do the test, the test was only conducted once; due to the high consistency and to ensure there was no training effect, most tests were not repeated numerous times. There was no time limit for completing each test, and the time to completion was recorded.

**Table 3.** Alternatives for the Forced-Choice Tests

Tests	Grating Visual Acuity	Tumbling E Visual Acuity	Direction of Motion
Forced choices	Horizontal	Up	Horizontal (left or right)
	Vertical	Down	Vertical (up or down)
	Diagonal left	Left	Diagonal left down to right up
	Diagonal right	Right	Diagonal left up to right down
			Diagonal right down to left up
			Diagonal right up to left down
Number of alternatives	4	4	8

## Results

### Clinical Safety

Three operations were conducted at the Xiangya Hospital, and two were at the Hainan Eye Hospital. The operations were conducted in two different hospitals by different surgeons, demonstrating that the implantation procedure can be performed by more than one surgeon.

**Figure 4.**
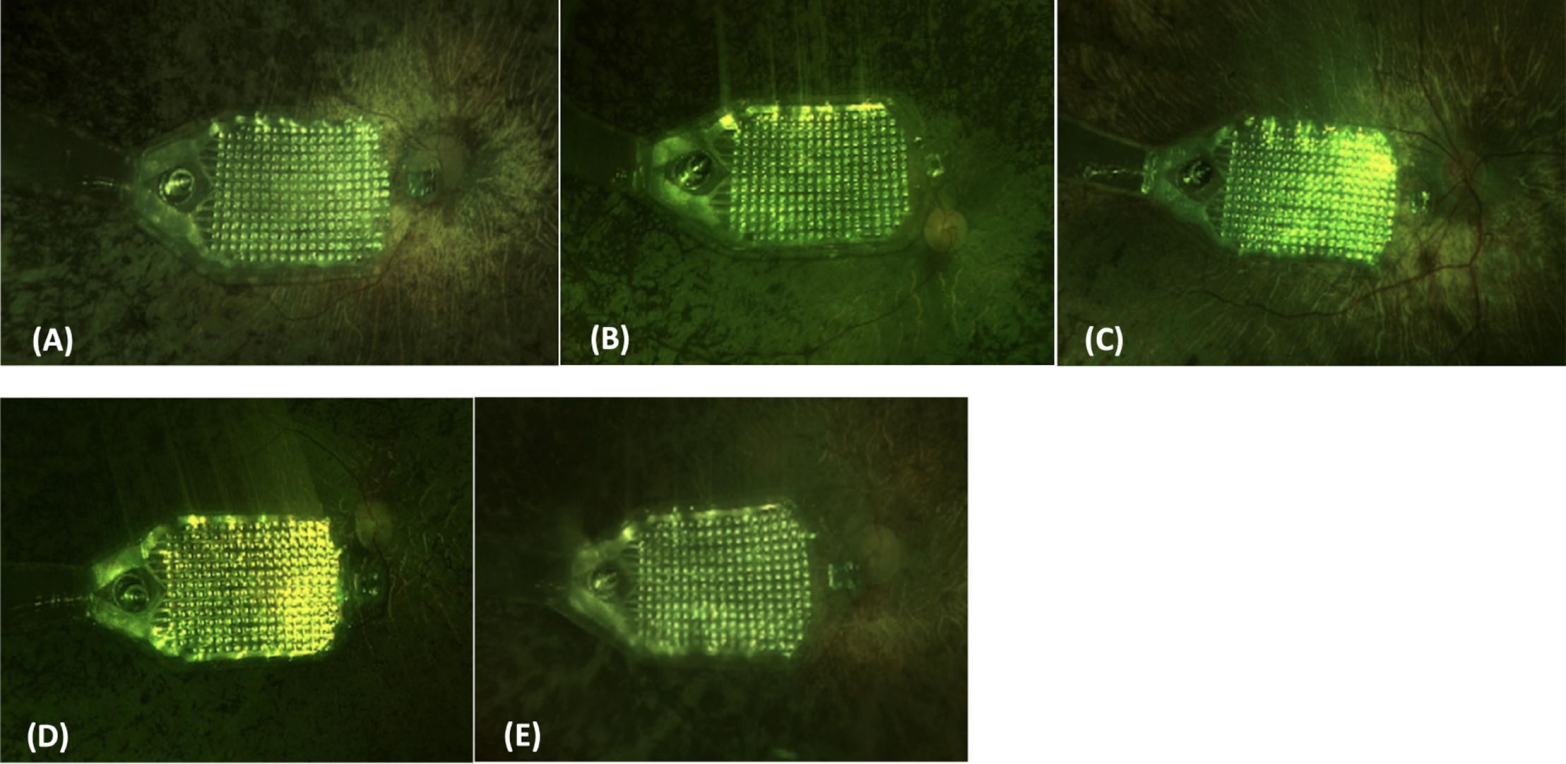
Ninety-day postoperative fundus photographs: (A) Subject P01, (B) Subject P02, (C) Subject P03, (D) Subject P04, and (E) Subject P05.

**Figure 5.**
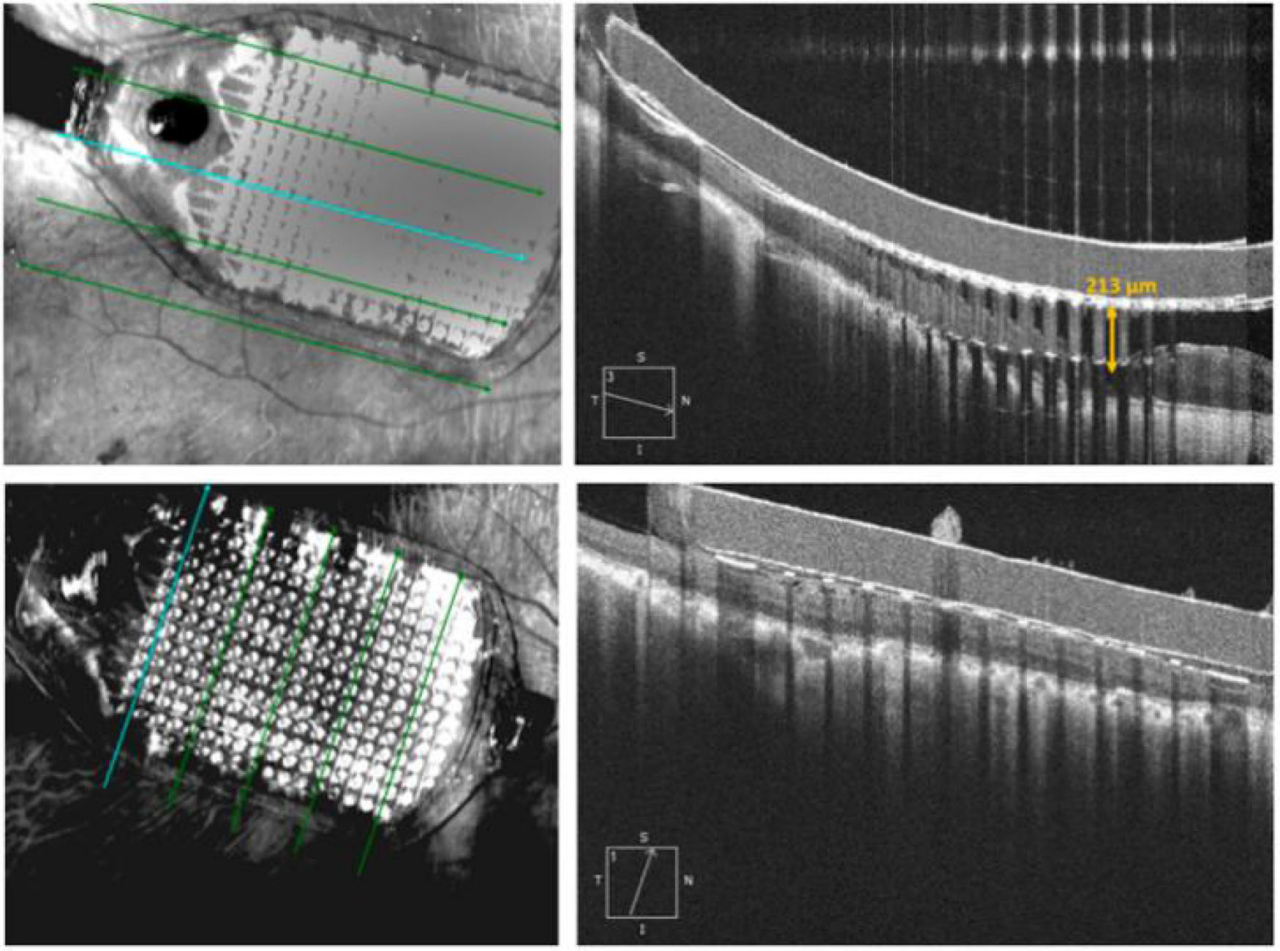
Ninety-day postoperative OCT images from Subject P03.

All five subjects had the IMIE 256 surgically implanted without complications. The average operation time was 180 minutes (170–190 minutes). All five IMIE 256 implant retinal electrode arrays were well positioned in the macular region, as shown in the postoperative fundus photographs (Fig. 4). The retinal electrode arrays were apposed to minimize the distance between the arrays and the underlying retina, as shown in the postoperative OCT images (Fig. 5). The distance from the median electrode to the internal limiting membrane ranged from 132 to 565 µm (Table 4).

**Table 4.** Electrode-to-Internal Limiting Membrane Distance

	Subjects				
	P01	P02	P03	P04	P05
Distance (µm), median (minimum–maximum)	542 (323–761)	248 (68–428)	132 (12–252)	313 (119–508)	565 (226–903)

The average IOP was 17.8 mmHg (range, 14–26) for the operated eye and 14 mmHg (range, 12–18) for the unoperated eye at postoperative 90 ± 10 days.

There were two occurrences of SAEs observed following implantation, and both events were in Subject P02.

During follow-up examination on postoperative day 3, the electrode array was observed to have moved slightly toward the upper temporal position in the macula. Hence, on postoperative day 5, Subject P02 underwent vitrectomy and repositioning of the electrode array. The electrode array remained stable and in a good position thereafter.

Subject P02 was also noted to have low IOP (<10 mmHg) in the implanted (right) eye 6 weeks after repositioning of the electrode array and hence at that time underwent repeat vitrectomy with resuturing of the scleral incision where the cable entered the vitreous cavity; silicone oil was used for tamponade. Thereafter the IOP normalized.

Other than the above two SAEs, all of the other subjects had expected uneventful postoperative healing of their incisions, and the implants remained stable in their position in both extraocular and intraocular locations.

The explantation surgeries were performed and there were no complications or SAEs related to explantation.

### Electrode Impedance and Threshold Current

The impedance of all 256 electrodes was measured using the quick impedance measurement function of the configuration system in the operation room and at the postoperative time points. As shown in Figure 1C, the retinal electrode array consists of 248 large electrodes (210 µm in diameter) and 8 small electrodes (160 µm in diameter). Electrodes were used in stimulation (effective electrodes) if their measured impedance value was less than 50 kΩ. Only one large electrode in one subject (P05) failed the 50-kΩ criterion, and the average number of effective small electrodes of the five subjects was eight, with an average impedance of 19.39 kΩ at the 90 ± 10 days postoperative time point, as shown in Table 5.

**Table 5.** Number of Effective Electrodes, Average Impedance, and Average Threshold Current Per Protocol Set at Postoperative 90 ± 10 Days

	Subjects					
Item	P01	P02	P03	P04	P05	Average of All 5 Subjects
Large electrodes (210 µm in diameter)						
Number of effective electrodes	248	248	248	248	247	247.8
Average impedance (kΩ)	7.71	11.78	26.68	12.26	11.48	13.98
Threshold current (µA), average (minimum, maximum)	383.6 (154, 400)	214.6 (88, 374)	101.4 (33, 308)	263.6 (132, 396)	219.8 (88, 396)	236.6 (33, 400)
Small electrodes (160 µm in diameter)						
Number of effective electrodes	8	8	8	8	8	8
Average impedance (kΩ)	13.22	27.08	27.58	15.61	13.47	19.39
Threshold current (µA), average (minimum, maximum)	400 (400, 400)	181.5 (121, 352)	140.3 (88, 198)	222.8 (132, 396)	264.0 (154, 396)	241.7 (88, 400)
Total number of effective electrodes	256	256	256	256	255	255.8
Average impedance of all effective electrodes (kΩ)	7.88	12.26	26.71	12.36	11.54	14.15

All subjects were able to perceive light (phosphenes) with controlled electrical stimulation when all 256 electrodes were turned on, except P05, who perceived light with 255 electrodes and had one electrode that was not functioning. The average threshold current for all five subjects’ large electrodes was 236.6 µA (range, 33–400); for all five subjects’ small electrodes, it was 241.7 µA (range, 88–400), as shown in Table 5.

The measured threshold currents for the five subjects showed a decreasing trend in subsequent sessions. The average threshold current and current range for all electrodes (*n* = 256) measured at time point 3 (90 ± 10 days) decreased compared to time point 1 (30 ± 10 days). As an example, Table 6 shows the average threshold currents of subject P03 in whom we performed a threshold current measurement at three postoperative time points (30 ± 10, 60 ± 10, and 90 ± 10 days).

**Table 6.** Average Threshold Currents of Subject P03

	Threshold Current (µA), Average (Minimum, Maximum)		
	30 ± 10 Days	60 ± 10 Days	90 ± 10 Days
Item	Postoperative	Postoperative	Postoperative
Large electrodes (210 µm in diameter) (*n* = 248)	129.7 (22, 400)	106.1 (33, 352)	101.4 (33, 308)
Small electrodes (160 µm in diameter) (*n* = 8)	170.1 (33, 400)	136.1 (44, 352)	140.3 (88, 198)
All electrodes (*n* = 256)	131.0 (22, 400)	107.1 (33, 352)	102.7 (33, 308)

In contrast to this decreasing trend in stimulation thresholds over months, the day-to day variability in threshold currents was minimal. Representative electrodes of each subject were chosen to repeat the threshold current test on each day for a maximum of 5 consecutive days. Three representative electrode stimulation threshold means and SD data of four subjects are shown in Table 7. The results show that there was little day-to-day variability in threshold currents, indicating that the retina–device interface was stable and the subjects were also reliable in their responses.

**Table 7.** Representative Electrode Stimulation Thresholds

	Subjects							
	P02		P03		P04		P05	
Representative Electrode	Threshold Current, Mean ± SD (µA)	No. of Tests	Threshold Current, Mean ± SD (µA)	No. of Tests	Threshold Current, Mean ± SD (µA)	No. of Tests	Threshold Current, Mean ± SD (µA)	No. of Tests
1	256.67 ± 20.74	3	58.67 ± 5.19	3	202.40 ± 16.46	5	149.60 ± 16.46	5
2	293.33 ± 10.37	3	44.00 ± 0.00	3	392.40 ± 9.33	5	242.00 ± 24.10	5
3	183.33 ± 20.74	3	51.33 ± 5.19	3	382.80 ± 10.78	5	154.00 ± 13.91	5

### Clinical Efficacy

All subjects completed the visual testing described in the Methods section through postoperative day 90.

#### Grating Visual Acuity

At the 90-day postoperative time point, all of the subjects performed better on the grating visual acuity test (0.025; 20/800) with the system on compared with both the system off and the baseline measurements (see Table 8).

**Table 8.** Grating Visual Acuity (Visual Acuity = 0.025) Evaluation Results Per Protocol Set

	Subjects									
	P01		P02		P03		P04		P05	
	System	System	System	System	System	System	System	System	System	System
Time Point	On	Off	On	Off	On	Off	On	Off	On	Off
Baseline, %	0		9		0		0		0	
90 ± 10 days postoperative, *n*	1/2	0/1	4/5	0/2	2/2	0/2	4/4	0/1	3/3	0/1
Average time (s)	28	9	27	10	32	8	18	10	22	12
Trials, *n*	2	1	5	2	2	2	4	1	3	1

#### Tumbling E Visual Acuity

At the 90-day postoperative time point, the subjects were able to achieve Tumbling E visual acuity of 0.017 (20/1200), whereas they were unable to do the Tumbling E test at baseline or with the system off (see Table 9).

**Table 9.** Tumbling E Visual Acuity (Visual Acuity = 0.017) Evaluation Results Per Protocol Set

	Subjects									
	P01		P02		P03		P04		P05	
	System On	System	System	System	System	System	System	System	System	System
Time Point	On	Off	On	Off	On	Off	On	Off	On	Off
Baseline, %	0		0		0		0		0	
90 ± 10 days postoperative, *n*	3/4	0/1	3/5	0/1	4/4^a^	0/1^a^	2/3	0/1	4/4	0/1
Average time (s)	95	28	98	12	75	14	120	20	69	24
Trials, *n*	4	1	5	1	4	1	3	1	4	1

a Postoperative 60 ± 10-day data were used because the subjects missed the 90 ± 10-day postoperative check.

#### Direction of Motion

Table 10 shows that all five subjects were able to identify the movement direction when the system was turned on. At the 90-day postoperative time point, all five subjects performed better with the system on compared with the system off or at baseline.

**Table 10.** Results of Direction of Movement Evaluation Per Protocol Set

	Subjects									
	P01		P02		P03		P04		P05	
	System	System	System	System	System	System	System	System	System	System
Time Point	On	Off	On	Off	On	Off	On	Off	On	Off
Baseline, %	0		0		0		0		0	
90 ± 10 days postoperative, *n*	3/4	0/1	4/4	0/1	3/3	0/1	4/4	0/1	4/4	0/1
Average time (s)	72	14	100	20	95	15	70	20	56	14
Trials, *n*	4	1	4	1	3	1	4	1	4	1

#### Square Localization

As seen in Table 11, at the 90-day postoperative time point, all of the subjects were able to localize the square light spot on the test screen with significantly improved accuracy with the system on compared with system off or at baseline.

**Table 11.** Square Localization Evaluation Results Per Protocol Set

	Subjects									
	P01		P02		P03		P04		P05	
	System	System	System	System	System	System	System	System	System	System
Time Point	On	Off	On	Off	On	Off	On	Off	On	Off
Baseline, %	0		0		0		0		0	
90 ± 10 days postoperative, *n*	4/5	0/1	4/4	0/1	3/3	0/1	4/4	0/1	2/2	0/2
Average time (s)	45	15	100	20	207	39	99	22	103	17
Trials, *n*	5	1	4	1	3	1	4	1	2	2

#### Orientation and Mobility

Four subjects (P01, P03, P04, and P05) were able to identify the door and the black line on the ground and maintain a straight trajectory without the aid of a cane. For both the line task and the door task, at 90 days after implantation the four subjects tested at 100% accuracy with the system on compared with 0% with the system off, as seen in Tables 12 and 13, respectively. Subject P02 has severe hearing loss, so it was not possible to instruct her to do these tests.

**Table 12.** Line Task Assessment Results Per Protocol Set

	Subjects									
	P01		P02		P03		P04		P05	
	System	System	System	System	System	System	System	System	System	System
Time Point	On	Off	On	Off	On	Off	On	Off	On	Off
Baseline, %	0		0		0		0		0	
90 ± 10 days postoperative, *n*	2/2	0/1	NA	NA	4/4^a^	0/1^a^	3/3	0/1	2/2	0/1
Average time (s)	82	16	NA	NA	75	22	72	29	66	27
Trials, *n*	2	1	NA	NA	4	1	3	1	2	1

a Postoperative 60 ± 10-day data were used because the subjects missed the 90 ± 10-day postoperative check. NA, no data available.

**Table 13.** Door Task Evaluation Results Per Protocol Set

	Subjects									
	P01		P02		P03		P04		P05	
	System	System	System	System	System	System	System	System	System	System
Time Point	On	Off	On	Off	On	Off	On	Off	On	Off
Baseline, %	0		0		0		0		0	
90 ± 10 days postoperative, *n*	2/2^a^	0/1^a^	NA	NA	2/2^a^	0/1^a^	1/1	0/1	1/1	0/1
Average time (s)	90	20	NA	NA	130	32	188	24	105	20
Trials, *n*	2	1	NA	NA	2	1	1	1	1	1

a Postoperative 60 ± 10-day data were used because the subjects missed this test at the 90 ± 10-day postoperative time point. NA, no test data available.

## Discussion

There are a number of other types of retinal implants that are placed subretinally and suprachoroidally. The Alpha IMS (Retina Implant AG, Reutlingen, Germany), a 1600-electrode photodiode array, is one such subretinal implant.^8^ A clinical trial reported that, in the first 12 months of observation, 13 out of 21 participants reported that restored visual function was useful for daily life, and visual acuity measured by Landolt C-rings reached up to 20/546.^9^ However, in spite of this early success, the Alpha IMS has now been abandoned. The Prima System (Pixium Vision, Paris, France), an intelligent retinal implant system, is another subretinal implant that is being tested in subjects with geographic atrophy in the setting of dry macular degeneration. Reported visual acuities have been in the range of 20/400 without zoom.^10^ Bionic Vision Technologies (Melbourne, Victoria, Australia) offers a suprachoroidal implant, and the best vision to date reported has been in the range of 20/4451 to 20/1059.^11^ The epiretinal implant with the most clinical experience is the Argus II. Hence, we have compared IMIE 256 to Argus II. The IMIE 256 was made possible by several breakthrough innovations in engineering to reduce the size of the electronic implant and yet increase the number of electrodes, with nearly 100% of electrodes functional from each manufacturing run.

The IMIE 256 is implanted in only one quadrant of the eye because of the smaller implant size; hence, the risk of exposure of the episcleral electronics capsule, which is one of the leading SAEs with Argus, is reduced (Argus is implanted in two quadrants and has an encircling silicone band).^1^ The flexibility of the cable and the highly contoured shape of the electrode array, matching the curvature of the retina, made the placement and subsequent tacking of the array to the retina easier. These aspects of the IMIE 256 have led to a reduction in SAEs. In the reported Argus studies, 18 SAEs were related to the device or the surgery in 10 out of 30 subjects 1 year after surgery and 23 SAEs in 11 out of 30 subjects 3 years later.^4^ In contrast, this study had two occurrences of SAEs in one out of five subjects. Although the number of subjects is smaller and the follow-up was shorter compared to the Argus studies, most of the SAEs in the Argus studies occurred in the first 90 days, which is the length of follow-up in this study. Therefore, there is some evidence that the IMIE 256 is easier and safer to implant.

Also, the greater density of functioning electrodes of the IMIE 256 implant resulted in the subjects performing better on a number of tests when compared with the Argus II. Out of the five IMIE 256 implants, all but one implants had 100% of the electrodes working, and the one implant had only one non-functioning electrode, yielding a collective success rate of 99.9% (1279/1280 functioning electrodes). This is an important achievement, as the average number of effective electrodes was around 94.4% in one Argus clinical study, and the Argus manufacturer only guaranteed 55/60 electrodes to be functional.^1^ On the grating visual acuity test, 27% to 48% of Argus II subjects scored between 2.9 and 1.6 logMAR at least once,^2^ whereas five of five subjects implanted with the IMIE 256 had a grating visual acuity of at least 20/800 (1.6 logMAR). In the Tumbling E visual acuity test, three of 30 Argus II subjects were able to recognize 1-inch characters at a viewing distance of 12 inches, representing a 20/1200 visual acuity, and 15 of 30 Argus II subjects were able to recognize 8.9-inch characters at a viewing distance of 12 inches, representing a 20/10206 visual acuity.^3^ In contrast, four of five IMIE 256 subjects could recognize 10.5-cm (4.13-inch) characters at a viewing distance of 1.2 m (47.24 inches), corresponding to a visual acuity of 20/1200. On the square localization and direction of motion tests, 96% and 57%, respectively, of subjects implanted with the Argus II, performed better with the system on versus off,^1^ whereas 100% of subjects implanted with the IMIE 256 performed better on both tests with the system on versus off. The mean percent difference between success rate with the system on versus the system off on the door task was 24% at 3 months for the Argus II^1^ compared with 80% for the IMIE 256. The mean percent difference on the line task was 48% for the Argus II,^1^ compared with 80% for the IMIE 256, albeit for this test the Argus II testing was performed with lines with turns.

Subjects also performed much better with the IMIE 256 system on versus system off or baseline. The median separation between the electrode and the retina increases at distances farther from the retinal tack, which possibly leaves room for lowering thresholds and improving visual performance with further improvement in design and surgical procedures to reduce this separation. In spite of instructions to “give their best effort for each test,” there is a possibility that subjects may not have put forth the same level of effort for the trials with the device off. This is not likely to have occurred in all subjects, and it would not have influenced the baseline measurements. However, we do plan to address this in future studies by developing a mode in which the device is on but the input from the camera is scrambled and not representative of the visual image.

For the IMIE 256, no intentional gross head scanning was observable during these tasks in order to combine the perceived information for a useful image, as was necessary with the Argus II. The charge required on both the large and small electrodes did not exceed the safety limits for neuronal stimulation, which bodes well for constructing an even higher density electrode array using only the smaller electrode size.^12^

The significantly reduced size and lower power consumption of the IMIE 256 reduces dissipated heat to the surrounding tissue due to a compact double telemetry coil design that improves power transmission efficiency and data transmission rate. Furthermore, a novel packaging technology employs multiple layers of biocompatible yet durable barrier material to withstand corrosion by bodily fluids for an extended period.^12^^–^^15^

Finally, the IMIE 256 implant uses a custom application-specific integrated circuit (ASIC) with a high lead-count connection technology that allows for reliable electrical connections between the ASIC and the 256 electrodes within a compact space. The high lead-count connection and multiple-layer packaging technologies improve the manufacturability for mass production, thus increasing the yield, and should reduce the cost of the device.

Although the results of the IMIE 256 are encouraging, this study is limited by the small sample size and shorter follow-up period as necessitated by various regulatory approvals for first in-human studies. Further testing to evaluate the IMIE 256 in more patients over a longer follow-up period is being planned.

## Supplementary Material

Supplement 1

## References

[1] Humayun MS, Dorn JD, da Cruz L, et al. Interim results from the international trial of Second Sight's visual prosthesis. *Ophthalmology**.* 2012; 119(4): 779–788. 22244176 10.1016/j.ophtha.2011.09.028 PMC3319859

[2] da Cruz L, Dorn JD, Humayun MS, et al. Five-year safety and performance results from the Argus II Retinal Prosthesis System clinical trial. *Ophthalmology**.* 2016; 123(10): 2248–2254. 27453256 10.1016/j.ophtha.2016.06.049 PMC5035591

[3] Lepri BP. H110002 Argus II Retinal Prosthesis Systems. Presentation to the Food and Drug Administration, Washington, DC, September 28, 2012.

[4] Ho AC, Humayun MS, Dorn JD, et al. Long-term results from an epiretinal prosthesis to restore sight to the blind. *Ophthalmology**.* 2015; 122(8): 1547–1554. 26162233 10.1016/j.ophtha.2015.04.032 PMC4516690

[5] Weiland JD, Humayun MS. Retinal prosthesis. *IEEE Trans Biomed Eng**.* 2014; 61(5): 1412–1424. 24710817 10.1109/TBME.2014.2314733 PMC4356127

[6] da Cruz L, Coley BF, Dorn J, et al. The Argus II epiretinal prosthesis system allows letter and word reading and long-term function in subjects with profound vision loss. *Br J Ophthalmol**.* 2013; 97(5): 632. 23426738 10.1136/bjophthalmol-2012-301525 PMC3632967

[7] Stronks HC, Dagnelie G. The functional performance of the Argus II retinal prosthesis. *Exp Rev Med Devices**.* 2014; 11(1): 23–30. 10.1586/17434440.2014.862494 PMC3926652 24308734

[8] Shapero A, Agarwal A, Martinez JC, Emami A, Humayun MS, Tai Y-C. Wireless implantable intraocular pressure sensor with parylene-oil-encapsulation and forward-angled RF coil. In: *2019 IEEE 32nd International Conference on Micro Electro Mechanical Systems (MEMS*). Piscataway, NJ: Institute of Electrical and Electronics Engineers; 2019: 21–24.

[9] Edwards TL, Cottriall CL, Xue K, et al. Assessment of the electronic Retinal Implant Alpha AMS in restoring vision to blind patients with end-stage retinitis pigmentosa. *Ophthalmology*. 2018; 125(3): 432–443. 29110946 10.1016/j.ophtha.2017.09.019 PMC5818267

[10] Palanker D, Le Mer Y, Mohand-Said S, Muqit M, Sahel JA. Photovoltaic restoration of central vision in atrophic age-related macular degeneration. *Ophthalmology*. 2020; 127(8): 1097–1104. 32249038 10.1016/j.ophtha.2020.02.024 PMC7384969

[11] Ayton LN, Blamey PJ, Guymer RH, et al. First-in-human trial of a novel suprachoroidal retinal prosthesis. *PLoS One*. 2014; 9(12): e115239. 25521292 10.1371/journal.pone.0115239 PMC4270734

[12] Shannon RV. A model of safe levels for electrical stimulation. *IEEE Trans Biomed Eng**.* 1992; 39(4): 424–426. 1592409 10.1109/10.126616

[13] Shapero AM, Liu Y, Tai Y-C. Parylene-on-oil packaging for long-term implantable pressure sensors. *Biomed Microdevices*. 2016; 18(4): 66. 27422106 10.1007/s10544-016-0089-4

[14] Shapero A, Liu Y, Tai Y-C. Parylene-on-oil packaging for implantable pressure sensors. In: *2016 IEEE 29th International Conference on Micro Electro Mechanical Systems (MEMS*). Piscataway, NJ: Institute of Electrical and Electronics Engineers; 2016: 403–406.

[15] Shapero A, Tai Y-C. Parylene-oil-encapsulated low-drift implantable pressure sensors. In: *2018 IEEE 31st International Conference on Micro Electro Mechanical Systems (MEMS)*. Piscataway, NJ: Institute of Electrical and Electronics Engineers; 2018: 47–50.

