# Supplementary material for: First Human Results With the 256 Channel Intelligent Micro Implant Eye (IMIE 256)
Source: Transl Vis Sci Technol. 2021 Oct 27;10(10):14. doi: 10.1167/tvst.10.10.14 (PMC8556567; doi:10.1167/tvst.10.10.14)
Supplement: Supplement 1 [file tvst-10-10-14_s001.pdf]

Supplementary Material

Document 1- Detailed Device Description

The IMIE™ 256 Implantable Device consists of two components: implant and retinal tack. The Implant has 3 parts: the episcleral electronic implant body, the flexible cable, and the retinal electrode array. The Implant has two different models; one for the left eye and one for the right eye. These models are identical except for the flexible cable, which has a different bent based on the eye type (OD/OS) to position the electrode array near horizontally in the macula. Moreover, each of the above models has two sizes. The G size implant has a longer flexible cable and a larger retinal electrode array radius for eyes with an axial length range of 23-26 mm compared to the M size implant, which is for eyes with an axial length of 20.5-23 mm.

The episcleral electronic implant body is designed to be sutured onto the sclera in the superotemporal quadrant, between the superior rectus and lateral rectus; and is under the tenons and conjunctiva. This implant body contains all the electronics components including a power coil for receiving the power; a data coil for bi-directional data transmission, an ASIC (Application-Specific Integrated Circuit) for the control of the implant electrical functions; discrete components for working with the ASIC. A custom multiple-layer packaging technology is used for a compact and reliable packaging design. The flexible cable, consisting of 256 microfabricated metal wires, runs through an incision in the sclera and connects the episcleral electronic implant to the retinal electrode array. The retinal electrode array consists of 256 microfabricated electrodes, which contact the ganglion side of the retina and delivers controlled stimulation current pulses to the retina. The retinal electrode array is microfabricated using a biocompatible polymer substrate - Parylene C. This polymer can be thermally reshaped to provide a 3D contoured shape for the retinal electrode array and hence a better fit to the

curvature of the retina.

The external subsystems contain a video capture and transfer unit (VCTU), a video processing unit (VPU), and a configuration/fitting system. In the Patient Mode, the VCTU acquires image data through two cameras situated on the wearable glasses. The main visible light video camera is a 350-950nm black and white sensor with an infrared filter, and the infrared camera can see the infrared spectrum within a 1-10μm wavelength range and provides a thermal image. The image signals are transmitted to the VPU, which is connected via a video and power cable to the VCTU. The VPU has a user interface (buttons) that allows for the subject to switch between the two separate cameras, or change settings such as mode, brightness, and zoom. Additionally, the VPU contains a low-power Bluetooth module for wireless communication with the configuration/fitting system. After the image data are processed and modulated according to the settings, they are sent back to the VCTU where they are wirelessly transmitted to the Implant via the external coil. The processed video image data are 15 levels (0-14) of grey scale of each electrode, and the maximum frame rate is 30 FPS. The power and data are received by the coils within the episcleral electronic implant body, where they are converted into electrical pulses by the ASIC and transmitted through the flexible cable to the retinal electrode array to stimulate the remaining retinal neurons. The reverse telemetry designed to communicate the measured voltage of selected electrodes for impedance calculation to the external system, which helps with ASIC power and ASIC temperature data.

Document 2- Implantation and explantation surgical details

The implantation surgical procedure is shown and described in Figure 6. Eight main surgical steps are included. A 270-degree conjunctival peritomy was performed and the rectus

muscles were isolated using 4-0 nylon (Ethicon, Somerville, New Jersey, USA). The crystalline lens was removed by phacoemulsification performed with a corneoscleral tunnel incision for Subjects P02, P03, and P04. Next, in all subjects, a 23-gauge pars plana vitrectomy was performed, followed by insertion of a scleral dissector in the superotemporal quadrant to create a pocket between the tenons and sclera. Then the electronic body of the implant was placed into this pocket. The implant body was sutured to the sclera using 5-0 nylon suture at 6-7mm posterior to the limbus (Ethicon, Somerville, New Jersey, USA). Next, a 6 mm circumferential full-thickness scleral incision was made 3-4.5 mm posterior to the limbus (in the area of the pars plana). The retinal electrode array and the flexible cable were inserted through this incision into the vitreous cavity. The incision was sutured with 7-0 vicryl sutures (Ethicon, Somerville, New Jersey, USA) avoiding damage to the cable. The retinal tack was inserted through the enlarged superonasal sclerotomy and used to affix the retinal electrode array on the retina in the macula. Then, all the sclerotomies were sutured with 7-0 vicryl (Ethicon, Somerville, New Jersey, USA), followed by using 7-0 vicryl to suture a partial-thickness donor sclera over the cable and implant body. The procedure concluded by removing the rectus stay sutures and suturing the conjunctiva and tenons using 6-0 plain gut (Ethicon, Somerville, New Jersey, USA)

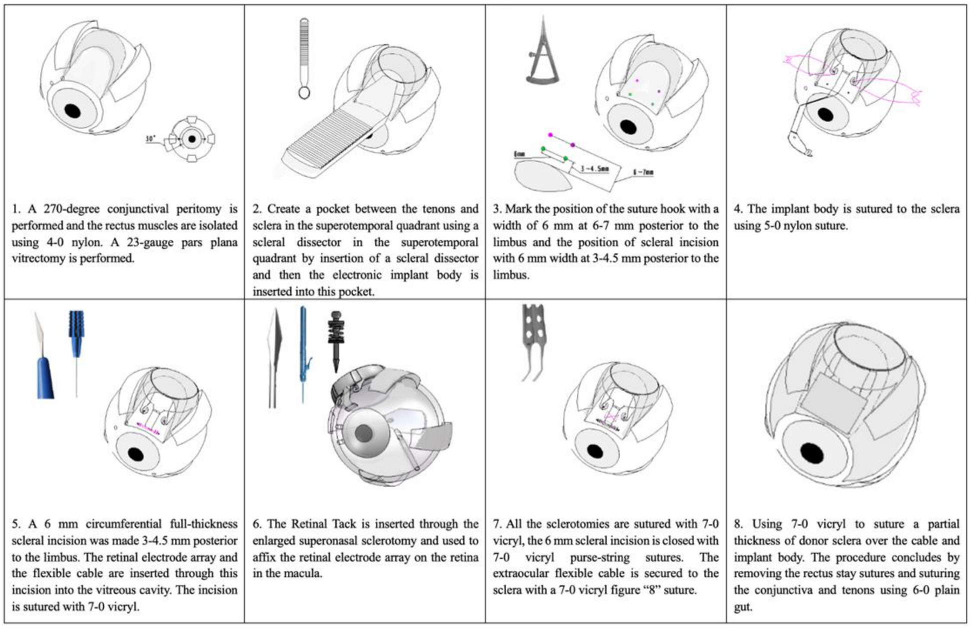

Figure 6: The IMIE^{TM} 256 Implantation Procedure.

The explanation surgical procedure is as follows: General anesthesia, sterile prep, and drape were the same as the pre-operative implantation procedure. Explantation also included conjunctival dissection, rectus muscle isolation, and establishing the pars plan trocar and cannulas. The retinal tack was removed with the same tack holder used during implantation. The sutures at the superotemporal incision securing the electrode cable were cut and the electrode array was removed, followed by re-suturing the incision. Next, any fibrous capsule around the body of the implant was dissected, followed by excision of the scleral patch graft, and the sutures holding the implant body were cut. Once the implant body had been freed from the ocular tissue, the remainder of the procedure was identical to implantation; sclerotomies were sutured, sutures used to isolate the rectus muscles were cut, the conjunctiva and tenons were sutured, and the periocular antibiotics and steroids were applied. Recommended postoperative medications were the same as post implantation excluding the use of oral steroids.

Document 3- Preoperative, perioperative, and postoperative medications

At the beginning of the surgery, 15 mg dexamethasone and 1 g cefazolin were injected intravenously. After the surgery, 1ml of cefazolin and 0.5 ml of dexamethasone solution were injected subconjunctivally, as well as an intravitreal injection of 0.1ml vancomycin and 0.1ml ceftazidime solution. Postoperatively, all subjects were given a regimen of 500 mg fluoroquinolone capsule b.i.d. for seven days, oral steroid prednisolone tablets; starting with 60mg and tapered off over 7 days, fluoroquinolone eye drops q.i.d. for seven days, topical tobramycin and dexamethasone eye drops q.i.d. for 7 days, topical prednisolone acetate eye drops q.i.d. for 30 days and then tapered off over the next 2 weeks, topical pranoprofen eye drops q.i.d. for 30 days, steroid prednisone ointment applied qd for 14 days, and 1 drop of 1% atropine gel qd for 14 days.
